# Editorial: Neurotechnologies and brain-computer interaction for neurorehabilitation

**DOI:** 10.3389/fnrgo.2023.1203934

**Published:** 2023-06-22

**Authors:** Athanasios Vourvopoulos, Mathis Fleury, Luca Tonin, Serafeim Perdikis

**Affiliations:** ^1^Department of Bioengineering, Institute for Systems and Robotics - Lisboa, Instituto Superior Técnico, Universidade de Lisboa, Lisbon, Portugal; ^2^Department of Information Engineering, Università degli Studi di Padova, Padua, Italy; ^3^Brain-Computer Interfaces and Neural Engineering Laboratory, School of Computer Science and Electronic Engineering, University of Essex, Colchester, United Kingdom

**Keywords:** brain-computer interfaces, EEG, neural interfaces, robotics, virtual reality, FES, neurorehabilitation, neuroergonomics

## 1. Scope

The field of neurotechnologies is rapidly advancing, and there is growing interest in developing brain interfacing systems that can enhance user interaction performance and improve acceptability (Greenberg et al., 2021). Neural interfaces have the potential to revolutionize the ways we interact with computer systems, such as through Brain-Computer Interfaces (BCIs). BCIs are communication systems that can provide an alternative non-muscular channel for explicit or implicit control of computer systems such as virtual reality (VR) (Vourvopoulos et al., 2019), robotic platforms (Tonin and Millán, 2021; Tonin et al., 2022), and word spellers (Fazel-Rezai et al., 2012). Mental-state monitoring is an example of a human-computer interaction (HCI) technology that enables the computer system to adapt to the user's cognitive or affective states (e.g., workload) like in passive BCIs (Zander et al., 2010; Lotte and Roy, 2019). However, there are still significant challenges that must be addressed before these technologies can be widely adopted, particularly in the rehabilitation domain where although brain diseases represent a major socio-economic burden worldwide, the translation of BCI solutions is still rather experimental (Feigin et al., 2021).

Recent longitudinal studies and case reports have shown the potential benefits of BCI-based therapies for motor rehabilitation (Cervera et al., 2018; Mane et al., 2020). Specifically, multimodal interventions utilizing BCI-aided robotic, VR, or Functional Electrical Stimulation (FES) training (Biasiucci et al., 2018) have yielded superior outcomes compared to traditional rehabilitation regimes (Cervera et al., 2018). Nonetheless, a commonly reported limitation in BCI is the inability of certain users to accurately control a BCI system (Allison and Neuper, 2010), resulting in poor skill acquisition during training (Jeunet et al., 2016; Perdikis and Millan, 2020).

The research presented here aims to contribute to the alleviation of these caveats and improve the user experience of BCI-actuated VR and robotic platforms. One key focus is on developing new techniques for more intuitive control and reduced training time so as to improve user performance and experience. Additionally, the works included here aim to improve the acceptability of neurotechnologies by reducing the users' cognitive load and by addressing user concerns related to safety and comfort.

## 2. Research highlights

In this Research Topic, we gather studies aspiring to improve user performance and acceptability of neurotechnologies (e.g., brain or muscle interfacing), and which could ultimately promote their translation into real-world applications.

With respect to the incorporation of VR into neurotechnologies, Amini Gougeh and Falk explore whether multisensory VR motor priming, where haptic and olfactory stimuli are present, can improve motor imagery (MI) detection performance in terms of accuracy and speed. Results showed that significant improvements in MI detection could be achieved, and an increasing modulation of brain activity was observed as stronger weights in the common spatial pattern filter. The authors suggest that multisensory motor priming prior to MI-BCI could improve detection efficacy. This is in line with prior research concerning the impact of VR as a way to passively prime the motor system preceding MI training (Vourvopoulos and Bermúdez i Badia, 2016), or actively through the use of FES (Kumari et al., 2022), or transcranial direct current stimulation (tDCS) (Chew et al., 2020). With regard to rehabilitation, Marin-Pardo et al. explore telerehabilitation with gamified VR tasks as a means for providing higher doses of repeated task-specific practice to restore upper limb function in chronic stroke patients. To address this issue, the authors present Tele-REINVENT which incorporates low-cost, portable electromyography (EMG) biofeedback that improves motor performance in stroke patients who can voluntarily perform muscle contractions, and who cannot currently benefit from direct BCI control. The system was used to reinforce the activity of the wrist extensor muscles while avoiding the coactivation of flexor muscles via computer games. The study also finds that all participants showed high adherence to the training protocol and reported enjoying using the system. Furthermore, in the context of studying the correlation between patient engagement and adherence to the treatment, Grevet et al. developed a model for BCI acceptability and distributed a questionnaire to a representative sample of the French general public. Results showed that BCIs were generally well accepted in the context of motor rehabilitation after stroke, with the perceived usefulness of the system being a major driver of behavioral intention. The authors suggest that their model and methodology could be adapted for use in future studies with different stakeholder groups, populations, and BCI applications.

Additionally, a number of articles in our Research Topic investigate the impact of visual perspective during robot motor imagery and observation, as well as the impact of focus and mental fatigue with applications in neuroergonomics and human-machine interaction. Specifically, Farabbi et al. show that the type of perspective (1st vs. 3rd person) may not influence the brain responses during an MI-BCI task for robotic hand control, with no significant differences over time between three consecutive sessions. While the type of perspective does not significantly affect brain responses during an MI-BCI task for robotic hand control, a first-person perspective generally results in stronger embodiment compared to a third-person perspective in terms of self-location and ownership (Toet et al., 2020). Further, the study by Hinss et al. proposes the use of mental state-based adaptive systems that can adapt the interaction between the operator and the interface to mitigate any detected degraded cognitive state, such as modifying information, presentation modality, stimuli salience, or task scheduling. The article suggests that promoting the application of mental state-based adaptive systems can be a safer and more efficient way of human-machine interaction. Concretely, mental state-based adaptive interface design is essential for success, drawing on cognitive science, human factors, and neuroergonomics (Ayaz and Dehais, 2021). Finally, Angioletti and Balconi assess the impact of interoceptive focus in electroencephalography (EEG); specifically, the ability to direct attention toward bodily sensations and to be aware of one's internal physical and emotional states. Results suggest that an EEG delta-alpha pattern emerges in temporo-central areas, indicating attention to visceral signals, particularly during interpersonal motor synchrony. Overall, little is known about how interoception impacts interpersonal synchronization mechanisms. Previous studies utilizing connectivity analysis have provided evidence for the specificity and dynamics of attentional mechanisms involved in interoception, highlighting the crucial role played by fronto-temporal widespread connections in characterizing post-feedback interoception (García-Cordero et al., 2017). This could have potential application in motor and/or cognitive interventions, such as in physiotherapy and logotherapy rehabilitation.

## 3. Summary

The collected articles confirm the potential of neurotechnologies such as brain and muscle interfaces to exert a significant impact on neuroergonomics and ultimately translate into restorative or assistive applications. However, limitations in usability and accessibility to BCI technology are still prominent. This is why further research in human-computer interaction, neuroengineering, and neuroergonomics is crucial for the design of robust BCI systems that can foster applicability breakthroughs. The anticipated advances will allow BCI technology to be accessible not only by patients but also by able-bodied users in domestic or professional environments, moving closer to the integration of neurotechnology with wearable systems and the Internet of Things. Consequently, future BCI research will enable a wide range of novel possibilities in the way users interact with a computer system (e.g., neuroadaptive interfaces), forming new and exciting interaction-design prospects.

## Author contributions

AV created the structure and initial draft. AV, MF, LT, and SP contributed the article summaries. All authors revised and approved the final version of the manuscript.
